# Effect of cancer-associated mutations in the PlexinB1 gene

**DOI:** 10.1186/1476-4598-11-11

**Published:** 2012-03-09

**Authors:** Chun Zhou, Oscar Gee-Wan Wong, John R Masters, Magali Williamson

**Affiliations:** 1 Prostate Cancer Research Centre, University College London, 67, Riding House Street, London W1W 7EJ, UK; 2 The Wistar Institute, 3601 Spruce Street, Philadelphia, PA 19104, USA; 3 Department of Pathology, The University of Hong Kong, HKSAR, China

**Keywords:** Plexin, Prostate cancer, Semaphorin, Rac, RhoD, ErbB2, c-Met

## Abstract

**Background:**

Semaphorins act as chemotactic cues for cell movement via their transmembrane receptors, plexins. Somatic missense mutations in the plexinB1 gene coupled with overexpression of the protein frequently occur in prostate tumours, indicating a role for plexinB1 in the pathogenesis of prostate cancer.

**Results:**

Two specific mutations found in prostate cancer enhance RhoD binding and one other mutation results in loss of inhibition of Rac-dependent Pak1 phosphorylation and lamellipodia formation and in impairment of trafficking of plexinB1 to the membrane. None of the three characterised mutations affect PDZRhoGEF binding, RhoA activity, the interaction of plexinB1with the oncogenes ErbB2 or c-Met or ErbB2 phosphorylation. The mutations have the net effect of increasing cell motility by blocking plexinB1-mediated inhibition of Rac while enhancing the interaction with RhoD, an anti-migratory factor.

**Conclusions:**

PlexinB1 mutations block plexinB1-mediated signalling pathways that inhibit cell motility.

## Background

Semaphorins are a group of 20 or more secreted or membrane bound proteins [1] that act as chemotactic cues for cells expressing their transmembrane receptors plexins [2]. Semaphorins affect cell behaviour in diverse ways, regulating cell motility [3], invasive capacity [4], adhesion [5] and cell and axon growth cone collapse [6]. Semaphorins consequently have a function in many physiological processes including angiogenesis [7], cell migration [8,9], immune regulation [10] and organogenesis, affecting nervous system [11,12], lung [13], kidney [14] and cardiovascular development [15,16] and epithelial-mesenchymal interactions [14]. The response of a cell to semaphorin stimulation depends on the type of responding cell and particular semaphorins can generate opposite reactions depending on cell type [17]. The transmembrane semaphorin receptors, plexins, either bind semaphorins directly, or in the case of most class 3 semaphorins, to a complex of neuropilins and plexins [2,18]. Semaphorin 4D (Sema4D) binds directly to its receptor, plexinB1 [2].

Semaphorin/plexin signalling results in activation of receptor tyrosine kinases and modulation of the actin cytoskeleton via regulation of several specific small RhoGTPases. PlexinB1 binds to RacGTP [19,20], sequestering it from its downstream effectors, such as Pak1 [21], and to Rnd [22] and RhoD [23]. Rac, Rnd and RhoD all bind to the same region in the cytoplasmic domain of plexinB1, the RhoGTPase binding domain (RBD) [23]. Rnd binding is required for the binding of R-Ras to plexinB1 and for the R-RasGTPase activating protein (GAP) activity of plexinB1 which inactivates R-Ras resulting in a decrease in integrin and PI3K activation [24,25]. Sema4D/plexinB1 signalling activates RhoA through activation of PDZRhoGEF and LARG which bind to the C-terminal of the plexinB1 protein [26]. PlexinB1 can also mediate the inhibition of RhoA via the recruitment of p190RhoGAP [27]. PlexinB1 interacts with the receptor tyrosine kinases c-Met [28] and ErbB2 [29] via their extracellular domains and Sema4D/plexinB1 signalling results in c-Met and ErbB2 phosphorylation [28,29]. Activated ErbB2 phosphorylates tyrosines on plexinB1 creating a binding site for PLCγ. PLCγ binding is required for Sema4D-mediated RhoA activation [30]. Sema4D can both promote and inhibit migration depending on the plexinB1 co-receptors expressed [31].

Crystal structures of the RBD [23] and of the cytoplasmic domain of plexinB1 in complex with Rac1 [32,33] have been determined. Bell et al. [33]., identified a second RhoGTPase binding site in addition to the RBD, adjacent to the Ras site, which stabilises a trimeric structure of plexinB1-Rac complexes.

We have previously found mutations in the plexinB1 gene in 8/9 prostate cancer bone metastases, 7/17 prostate cancer lymph node metastases and 41/89 primary cancers, together with overexpression of the protein [34]. The mutations in plexinB1 enhance adhesion, migration and invasion *in vitro *and inhibit cell collapse [34]. The finding of functionally significant mutations in plexinB1 in prostate tumours and overexpression of the plexinB1 protein suggests that plexinB1 has a role in prostate cancer and so is a potential target for therapy. However, the mechanism by which plexinB1 contributes to prostate cancer progression is yet to be determined.

## Results

### Mutations in plexinB1 increase RhoD binding

**Figure 1:**
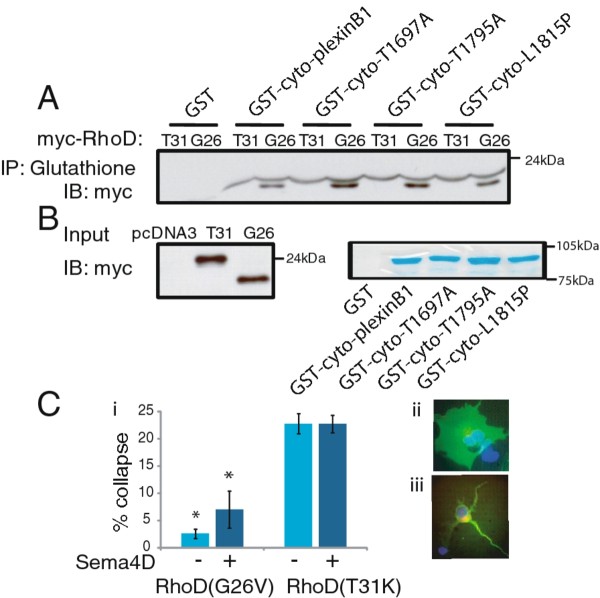
**Mutation of plexinB1 enhances RhoD binding**. A).GST-cyto-plexinB1 (WT and mutant) fusion proteins were used in GST pulldown assays with lysates of HEK293 cells expressing constitutively active (G26V) or dominant negative (T31K) RhoD-myc. B). Input of RhoD(G26V)-myc, RhoD(T31K)-myc and WT and mutant GST-cyto-plexinB1 fusion proteins. C). Expression of RhoDGTP decreases cell collapse. COS-7 cells co-transfected with plexinB1, Rnd and RhoD(G26V)-myc, or RhoD(T31K)-myc were stimulated with sema4D for 5mins, fixed and stained for plexinB1 (FITC) and myc (TRITC) and the % cell collapse scored. i).% cell collapse +/- SE, * *p *< 0.001 versus RhoD(T31K) 2 tailed ttest. ii) representative non-collapsed cell. iii) representative collapsed cell.

RhoD binds to the RBD domain of plexinB1 in the region where mutations are found in prostate cancer [35]. To determine if the mutations affect binding of RhoD to plexinB1 we performed GST pull down assays using GST-cyto-plexinB1(wild type (WT) or mutant) fusion proteins and dominant negative (T31K) or constitutively active (G26V) RhoD. Three mutations were characterised in these experiments: A5359G (amino acid change T1697A), A5653G (amino acid change T1795A) and T5714C (amino acid change L1815P). Constitutively active RhoD(G26V) bound to the cytoplasmic domain of WT plexinB1, but dominant negative RhoD(T31K) did not (Figure 1). Binding of RhoDGTP to plexinB1 was not inhibited by the mutations in plexinB1. The interaction was enhanced by the presence of mutations A5359G (T1697A) and A5653G (T1795A) in the cytoplasmic domain of plexinB1 (Figure 1). To test the effect of RhoDGTP expression on plexinB1 function, we performed collapse assays on COS-7 cells co-transfected with plexinB1, Rnd, and RhoD(G26V) or RhoD(T31K). Expression of constitutively active RhoD significantly reduced cell collapse (Figure 1C).

### Mutation of plexinB1 results in loss of inhibition of RacL61-dependent Pak1 phosphorylation

**Figure 2:**
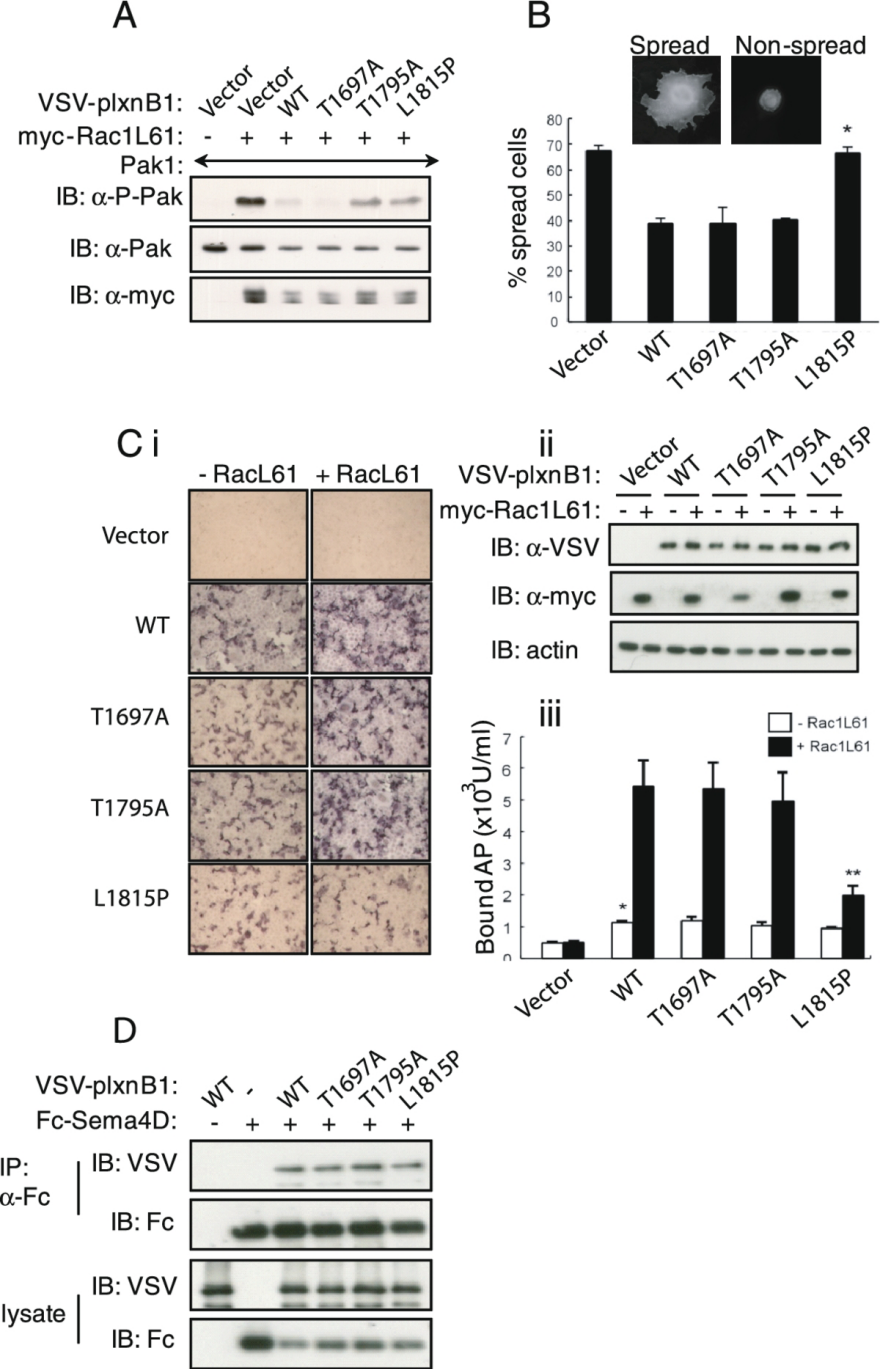
**The effect of plexinB1 mutations on Rac signalling**. A). Mutation of plexinB1 impairs inhibition of Pak1 phosphorylation. The phosphorylation status of Pak1 in COS-7 cells transfected with plexinB1 (WT or mutant), RacL61 and Pak1 was monitored by western blotting using anti- phospho199/204-Pak1 antibody. B). Mutation of plexinB1 impairs inhibition of cell spreading. COS-7 cells transfected with RacL61 and plexinB1 (WT or mutants) were plated on fibronectin for 2 h and those with a large surface area and discernible lamellipodia ('spread' phenotype) scored. Bars represent means +/- SE, *p < 0.05 versus RacL61 + WT, one tail *t*-test. C). Mutation in plexinB1 inhibits Rac-dependent trafficking of plexinB1 to the cell surface. COS-7 cells co-transfected with plexinB1 (WT or mutant) and RacL61 were treated with Sema4D-AP fusion protein and cell surface binding of Sema4D-AP assessed. i). Sema4D-AP cell surface binding detected with BCIP/NBT solution, representative of 3 independent experiments, ii). Western blot to show expression of VSV-plexinB1 and myc-RacL61 in transfected cells. iii). Bound alkaline phosphatase activity in transfected cells. Bars represent means +/- SE. *p < 0.01 versus vector without RacL61; **p < 0.01 versus WT with RacL61, one tail *t*-test. D). Mutation of plexinB1 does not affect Sema4D binding. HEK293T cells were cotransfected with VSV-plexinB1 (WT or mutant) and Sema4D-Fc. The plexinB1 protein in the cell lysate was immunoprecipitated with anti-Fc antibody.

PlexinB1 on the plasma membrane binds to and sequesters Rac-GTP and inhibits it from activating downstream effectors such as p21-activated kinase1 (Pak1) [21]. We have previously found that binding of Rac1 to the cytoplasmic domain of plexinB1 is inhibited by L1815P and is diminished by T1795A and T1697A amino acid changes [34]. To test the effect of cancer-associated mutations of plexinB1 on Rac-dependent Pak1 phosphorylation, WT plexinB1 or three different mutant forms of the protein were co-expressed with RacL61 and Pak1 in COS-7 cells. The phosphorylation status of Pak1 was monitored by western blotting using anti-phospho199/204-Pak1 antibody. As previously reported [36] coexpression of Rac1L61 and Pak1 stimulated the autophosphorylation of Pak1 (Figure 2A). Co-expression of WT plexinB1 with Rac1L61 and Pak1 inhibited Pak1phosphorylation. In contrast co-expression of plexinB1 (T1795A) or plexinB1 (L1815P) with Rac1L61 and Pak1 did not inhibit Pak1 phosphorylation. PlexinB1 (T1697A) inhibited Rac-dependent Pak1 phosphorylation to a similar extent as WT. Therefore the T5714C(L1815P) and A5653G (T1795A) mutations in plexinB1 result in loss of plexinB1-mediated inhibition of Rac-dependent Pak1 phosphorylation.

### Mutation of plexinB1 results in loss of inhibition of RacL61-dependent cell spreading

Cell spreading and extension of lamellipodia are phenotypes characteristic of Rac1 activation. COS-7 cells transfected with RacL61 exhibit a "spread" phenotype with a large surface area and discernible lamellipodia 2 h after plating on fibronectin (Figure 2B). Expression of WT plexinB1 reduced the number of cells showing this phenotype, consistent with the binding and sequestration of RacGTP by plexinB1, which effectively prevents Rac1 from participating in other signaling pathways. The inhibitory effect on cell spreading was retained in cells expressing plexinB1 (T1697A) and (T1795A). In contrast, the inhibitory effect on cell spreading was lost in cells expressing plexinB1 (L1815P).

### Mutation of plexinB1 inhibits Rac-dependent trafficking of plexinB1 to the cell surface

Rac1-GTP binds to and facilitates the cell surface expression of plexinB1 [21]. We sought to determine if mutation of plexinB1 affects Rac-dependent cell surface trafficking of plexinB1.

COS-7 cells co-transfected with plexinB1 (WT or mutant) and RacL61 were treated with Sema4D-AP fusion protein and cell surface binding of Sema4D-AP assessed. In the absence of RacL61 co-expression, COS-7 cells expressing WT or three mutant forms of plexinB1 showed similar levels of Sema4D-AP cell surface binding, significantly higher than cells not transfected with plexinB1 (*p *< 0.01) (Figure 2C). Sema4D-AP cell surface binding was enhanced when RacL61 was co-expressed with WT plexinB1. Similarly, cell surface binding of Sema4D-AP to COS-7 cells was enhanced when RacL61 was co-expressed with plexinB1(T1697A) and plexinB1(T1795A) to a similar extent as WT. In contrast, co-expression of RacL61 with plexinB1 (L1815P), did not enhance cell surface binding of Sema4D-AP. A significant difference in staining intensity was observed between cells expressing WT and plexinB1(L1815P) in the presence of Rac1L61 (*p *< 0.01) but not in the absence of Rac1L61. As the Sema4D binding capacity of plexinB1 was not affected by the mutations (as shown by immunoprecipitation, Figure 2D), our results show that the L1815P change inhibits Rac-dependent trafficking of plexinB1 to the cell surface.

Taken together, these results show that the T5714C (L1815P) mutation renders plexinB1 defective in sequestering and inhibiting Rac1 function and in facilitating plexinB1 trafficking. The A5653G (T1795A) mutation, which reduces RacGTP binding, also failed to inhibit Pak1 activation, although no discernible effect on cell spread or trafficking was seen.

### Effect of mutations on RhoA activation

**Figure 3:**
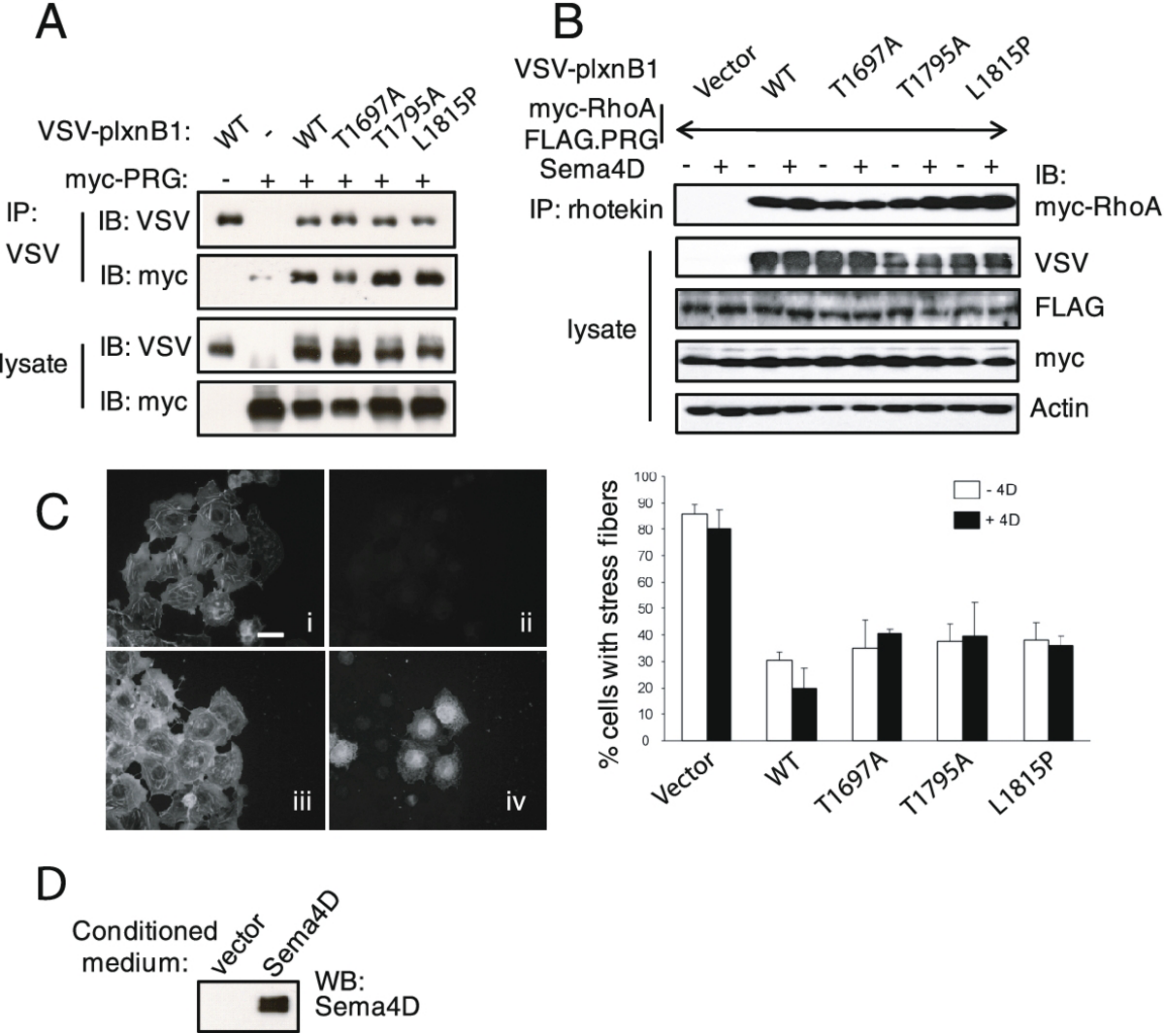
**Effect of plexinB1 mutation on RhoA activation**. A). Mutation of plexinB1 does not affect PDZRhoGEF (PRG) binding. Lysates of HEK293 cells co-expressing VSV-plexinB1 and PDZRhoGEF were immunoprecipitated with anti-VSV and bound proteins detected by western blotting. B). Mutation of plexinB1 does not affect RhoA activation. Lysates of HEK293 cells co-expressing VSV-plexinB1, FLAG-PDZRhoGEF and myc-RhoA were immunoprecipitated with GST-Rhotekin and RhoGTP detected by western blotting with anti-myc. C). COS-7 cells seeded onto coverslips were either transfected with plexinB1 (iii and iv) or vector (i and ii). Fixed cells were stained with anti-plexinB1 (ii and iv) or for actin (i and iii). Panel i and ii, and iii and iv, show the same field. Scale bar = 20 μm. D). The number of cells possessing more than three straight actin bundles of at least 5 μm long was counted and expressed as a percentage of the total number of cells in the field. Bars represent means +/- SE. D). Sema4D in conditioned medium. Conditioned medium was collected from Cos7 cells transfected with vector or Sema4D-Fc and the protein detected by immunoblotting with anti-Sema4D antibody.

PlexinB1 binds to the RhoGEFs, PDZRhoGEF and LARG, resulting in activation of RhoA. We investigated whether the mutations affect the interaction between PDZRhoGEF and plexinB1 and plexinB1-mediated Rho activation. Immunoprecipitation of PDZRhoGEF with anti-VSV from lysates of HEK293 cells co-expressing PDZRhoGEF and VSV-plexinB1 indicated that the mutations have little effect on PDZRhoGEF binding to plexinB1 (Figure 3A). Rho activation assays, using rhotekin pull down which binds to active RhoGTP selectively, showed that mutant forms of plexinB1 activate Rho to a similar extent as the WT form of plexinB1(Figure 3B). Expression of exogenous plexinB1 (WT or mutant) resulted in Rho activation in both the presence and absence of Sema4D, showing that overexpression of the plexinB1 receptor can mimic ligand binding as shown before [28]. Therefore the mutations do not affect plexinB1-mediated Rho activation.

PlexinB1 can also inhibit Rho activation via its interaction with p190RhoGAP [27]. Expression of WT plexinB1 inhibits the formation of stress fibres (an indicator of Rho activity) in COS-7 cells (Figure 3C), suggesting that RhoA is inhibited by plexinB1 in these cells, possibly via p190RhoGEF. The ability of three plexinB1 mutants to inhibit stress fibre formation was not significantly different from that of WT (Figure 3D). Taken together we saw no evidence that the three mutant forms of plexinB1 tested affect RhoA signalling.

### Effect of mutations on the interaction of ErbB2 and c-met with plexinB1 and on plexinB1-mediated phosphorylation of ErbB2

**Figure 4:**
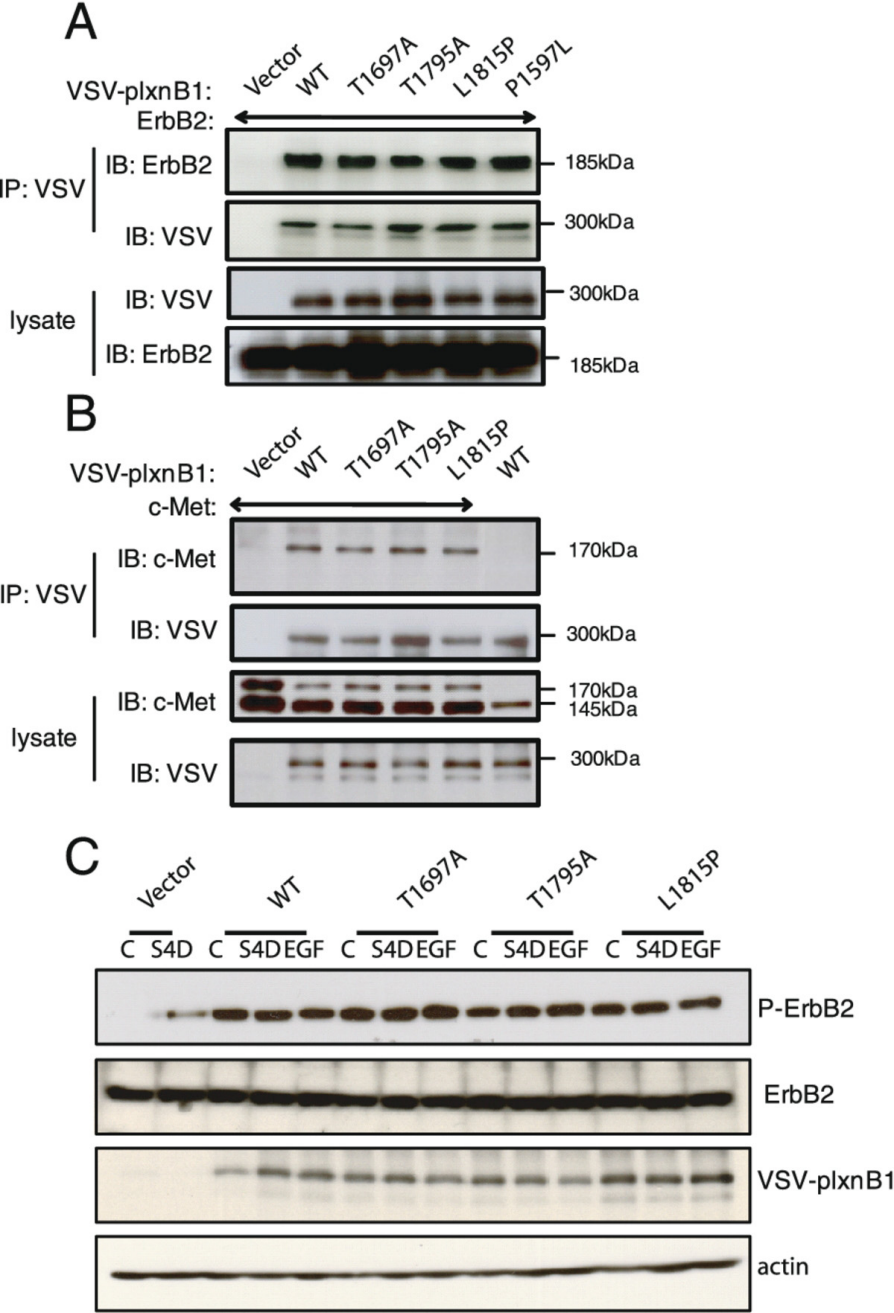
**Mutation of plexinB1 does not affect interaction of plexinB1 with ErbB2 or c-Met**. Lysates of HEK293 cells co-expressing ErbB2(A) or c-Met (B) and VSV-plexinB1 (WT or mutant) were immunoprecipitated with anti-VSV and bound proteins detected with anti-ErbB2 (A) or anti c-Met (B). Representative of 3 independent experiments. C). HEK293 cells transfected with plexinB1(WT or mutant) or vector were treated with control or sema4D conditioned medium or with EGF(500 ng/ml) for 20 mins and the phosphorylation of ErbB2 detected by immunoblotting.

PlexinB1 interacts with and activates the receptor tyrosine kinases ErbB2 [29] and c-Met [28] and Sema4D stimulation results in phosphorylation of both proteins in transfected HEK293 cells [31]. To determine if mutations of plexinB1 affect the interaction of plexinB1 with ErbB2, HEK293 cells were co-transfected with VSV-plexinB1(WT or mutant) and ErbB2. ErbB2 was co- immunoprecipitated with anti-VSV (Figure 4A). Both WT and mutant forms of plexinB1 interacted with ErbB2 and the strength of interaction between the different mutant forms of plexinB1 and the WT form were comparable. Similarly, all mutant forms of plexinB1 tested bound to c-Met to an equivalent extent as WT plexinB1 in cells co-expressing VSV-plexinB1 (WT or mutant) and c-Met (Figure 4B). WT and mutant forms of plexinB1 pulled down the 170 kDa extracellular form of c-Met indicating that different forms of plexinB1 interact with the extracellular domain of c-Met. Therefore mutations in plexinB1 do not affect the interaction of plexinB1 with ErbB2 or c-Met. Exogenous expression of WT plexinB1 increased phosphorylation of ErbB2 (Figure 4C), and the mutations had little effect on this process.

## Discussion

Mutations in the gene for plexinB1 are frequent in prostate cancer and the plexinB1 protein is overexpressed in prostate tumours, indicating that plexinB1 has a role in prostate cancer. The aim of this study was to investigate the mechanism by which mutation of plexinB1 contributes to prostate cancer progression. The three different cancer-associated plexinB1 mutations that were investigated in this study affect plexinB1-small RhoGTPase signalling in different ways, but have no effect on ErbB2, c-Met binding or RhoA activity.

Surprisingly, in spite of its position in the RBD region of plexinB1, the L1815P change does not impair RhoD binding. Rac has been shown to bind to a second site close to the R-Ras binding site in the cytoplasmic domain of plexinB1, in addition to the RBD site [33]. It is possible that RhoD also binds to this second site (site B in reference [33]) so that mutations in the RBD would not prevent RhoD binding. Alternatively the mode of binding of RhoD to the RBD may be different from that of the Rnd1 and Rac1. In an analogous way, the L1815P change inhibits Rnd1 binding to the RBD but not Rnd2 binding [37]. Binding of RhoDGTP to plexinB1 is also retained by the T1795A and T1697A mutant forms, and binding is enhanced by these mutations. The T1795A amino acid change occurs within the RBD region and the T1697A change occurs outside of this region [35].

All 3 mutant forms have lost R-RasGAP activity, yet only the L1815P mutation inhibits Rnd binding. Loss of Rnd1 binding would account for the loss of R-RasGAP activity in the L1815P mutant form, since R-RasGAP activity is dependent on Rnd binding [24]. In contrast, the T1795A and T1697A forms bind Rnd, yet R-RasGAP activity is lost. Interestingly, RhoD has been shown to antagonise Rnd in signalling via plexinA1 [38]. We have found that RhoDGTP reduced cell collapse when co-expressed with Rnd and plexinB1. If RhoD does antagonise Rnd signalling via plexinB1, the increase in RhoD binding seen in the T1795A and T1697A mutant forms may contribute to the loss of R-Ras GAP activity seen for these forms.

RhoDGTP inhibits stress fibre formation and motility [39]. Binding of RhoDGTP to WT plexinB1 may sequester RhoD from downstream signalling partners. Expression of the T1697A and T1795A mutant forms of plexinB1 in tumour cells is predicted to have the effect of releasing the cell from the inhibitory effect of RhoD on motility and stress fibre formation, thereby promoting motility.

RacGTP binds and activates the autophosphorylation of Pak1 [36]. WT plexinB1 binds and sequesters RacGTP and thereby inhibits Pak1 phosphorylation [21]. Phosphorylation of Pak1 is not however inhibited by co-expression of RacGTP with the L1815P or T1795A mutant forms of plexinB1. The L1815P sequence change inhibits Rac, Rnd and R-Ras binding and the intrinsic R-RasGAP activity of plexinB1 [34]. The T1795A mutation does not affect Rnd binding but reduces Rac binding and inhibits R-Ras binding and R-RasGAP activity [34]. The release of inhibition of Pak1 phosphorylation by tumour cells expressing the L1815P and T1795A mutant forms of plexinB1is expected to result in an increase in MAPK signalling and to promote tumour progression.

Activation of Rac results in reorganisation of the actin cytoskeleton to form lamellipodia at the leading edge of migrating cells, a phenotype that is inhibited by WT plexinB1 expression. The L1815P mutation in plexinB1 blocks this inhibition of lamellipodia formation. The release from lamellipodia inhibition is expected to result in an increase in motility, and is consistent with the increase in motility we have observed in cells expressing this mutant form of plexinB1 [34]. We have previously shown that overexpression of the three characterised mutations in HEK293 cells, without exogenous RacL61 expression, resulted in an increase in cell spreading above that of vector controls [34]. These results suggest that in addition to the loss of inhibition of Rac function shown here, the mutations confer a gain of function which results in an increase in cell spreading.

Rac has also been shown to act upstream of plexinB1 facilitating the trafficking of plexinB1 to the cell membrane. Cells expressing the L1815P mutated form of plexinB1 show a decrease in cell surface expression of the protein.

## Conclusions

Activated plexinB1 functions either as a positive or negative regulator of several signalling pathways that promote cell migration. ErbB2, c-Met and RhoA are activated by plexinB1, enhancing cell migration. In contrast, Rac, Rnd and R-Ras are inhibited by plexinB1, resulting in a decrease in cell motility. The response of a cell to plexinB1 activation depends on a balance between these signaling pathways. The mutations we identified have no effect on ErbB2, c-Met or PDZRhoGEF binding or RhoA activity which enhance migration, but one or more of the mutations inhibit or hinder Rac, Rnd, and R-Ras binding and R-RasGAP [34] activity. The mutations thus have the net effect of increasing cell motility by the loss of inhibitory pathways. Two of the mutations also promote sequestration of RhoDGTP, an anti-migration factor.

## Methods

### Plasmid constructs and cell culture

The expression constructs for VSV-plexinB1and Sema4D-AP were kind gifts from Dr. L. Tamagnone. The region on plexinB1 cDNA encoding the intracellular domain (amino acids 1512-2135, accession no. X87904) was amplified by PCR and cloned into pGEX-4 T-3 (Amersham) using SalI and XhoI sites to produce pGEXB1cytoWT. The prostate cancer-associated mutations: L1815P, T1795A and T1697A were introduced into pGEXB1cytoWT and into VSV-plexinB1 by using QuikChange II XL *in vitro *mutagenesis kit (Stratagene). Other constructs were kindly provided by the following: Dr. KL Guan, pRK5-mycRacL61 and N17; Professor Takeshi Endo (Chiba University, Japan): RhoD pEF-BOS/Myc-RhoD G26V and T31K; Dr. Hitoshi Kikutani (Osaka University, Japan): Sema4D-Fc; Dr. J. Swiercz (Heidelburg): FLAG-PDZRhoGEF, ErbB2, RhoA; Dr M. Driessens: myc-PDZRhoGEF; Prof. J.Chernoff (Fox Chase Cancer Centre): pCMV-myc-hPak1. HEK293 and COS-7 cells were grown in DMEM (10% FCS).

### Antibodies

The following antibodies were used: anti-VSV, anti-FLAG, anti-myc (Sigma, V4888, F7425, C956), anti-Human IgG Fc (Jackson ImmunoResearch, 109-005-098), anti-β-actin (Abcam, ab6276), anti-Pak1 (Chemicon, AB3844), anti-P-Pak, anti-phospho-ErbB2 (Cell Signalling), ErbB2 (Millipore), Sema4D and plexinB1 (ECM Biosciences), c-Met (c-28), plexinB1 (H300), (Santa Cruz).

### Rac-mediated Pak1 phosphorylation

COS-7 cells were transfected with Pak1, constitutively active Rac (RacL61), and plexinB1(WT or mutant) or empty vector using Lipofectamine (Invitrogen). Lysates were analysed for Pak phosphorylation using a phospho-Pak1 antibody.

### Recombinant Sema4D

COS-7 cells transfected with Sema4D-Fc or Sema4D-AP or empty vector (control) were grown in serum free medium for 72 h. The conditioned medium was collected and used directly or purified. Sema4D concentration was assessed by western blotting (Figure 3).

### Cell spread assays

COS-7 cells transfected with myc-RacL61 and VSV-plexinB1 (WT or mutant) or vector were plated on 10 μg/ml fibronectin. Unattached cells were washed away after 2 h. Attached cells were fixed (4% paraformaldehyde), permeabilized (0.1% Triton-X 100), blocked (2% BSA) and subjected to immunofluorescence staining (anti-myc). At least 200 myc-RacL61 positive cells were scored per slide.

### *In situ *binding of Sema4D-AP with plexinB1

COS-7 cells transfected with plexinB1 (WT or mutant) and RacL61 or vector were washed with HBAH (Hanks balanced salt solution with 20 mM HEPES pH 7.0, 0.5 mg/ml BSA, 0.1% (w/v) NaN3), treated with HBAH containing 1,000 ng/ml Sema4D-AP for 90 min then washed with ice-cold HBAH and fixed (65% (v/v) acetone, 8% (v/v) formalin, 20 mM HEPES pH 7.0). The cells were incubated at 65°C for 100 min, then in BCIP/NBT solution in the dark.

### Immunoprecipitation

Lysates of HEK293 transfected cells were incubated with 1 μg of selective antibody for 2 h at 4°C. The antigen-antibody complex was incubated with Protein-G sepharose for 2 h, washed 3 times and analyzed by immunoblotting.

### GST-pull down assay

Lysates of COS-7 cells expressing RhoDG26V or RhoDT31K were incubated with GST-fused cyto-plexinB1 (WT or mutant) overnight. Following washing of the matrix, the samples were analysed by SDS-PAGE and immunoblotting.

### Collapse assay

COS-7 cells plated on coverslips were co-transfected with plexinB1, Rnd and RhoDG26V-myc or RhoDT31K-myc. 48 h post transfection, the cells were treated with sema4D (4 μg/ml) for 5 mins, then fixed with 4% paraformaldehyde, permeabilised with 0.2% triton, then stained by immunofluorescence using anti-plexinB1 antibody(R&D) (FITC secondary antibody(Southern Biotech)) and anti-myc antibody (Abcam) (TRITC secondary). Co-transfected cells were sized using imageJ and cells with a size of < 500 μm2 with 3 or more processes were scored as 'collapsed'. Cells were scored 'blind'. The experiment was performed 3× in triplicate with a total of 186 or more co-transfected cells counted per condition.

### Rho activity assay

HEK293 cells were transfected with RhoA, PDZRhoGEF and plexinB1 (WT or mutant) or vector control. 48 h after transfection, cells were treated with control or Sema4D conditioned medium for 25 mins and the lysate incubated with 50 μl Rhotekin (Upstate) for 1 h, washed 3 times and the pulled down protein analysed by western blot with anti-myc antibody.

### Stress fibre assay

COS-7 cells transfected with plexinB1 (WT or mutant) were fixed (4% paraformaldehyde), permeabilized (0.1% Triton-X 100), blocked with 2% BSA then subjected to immunocytochemistry with anti-plexinB1 (SantaCruz) then goat-anti-mouse FITC (SouthernBiotech) supplemented with Phalloidin-TRITC (Sigma). The number of cells possessing more than three straight actin bundles of at least 5 μm long was counted. The identities of the slides counted were blinded to the researcher. At least 50 cells were counted per slide.

### PlexinB1-mediated ErbB2 phosphorylation

HEK293 cells transfected with vector or plexinB1 (WT or mutants) were serum starved over 2 nights then treated with control or sema4D conditioned medium or rhEGF(500 ng/ml, R&D Biosytems) for 20 mins. Lysates were analysed for ErbB2 phosphorylation using an anti phospho-ErbB2 antibody.

## Competing interests

A patent has been filed by M.W. and J.R.M. for mutations.

## Authors' contributions

CZ and OW carried out the molecular genetic studies. JM participated in its design and coordination. MW conceived of the study, coordinated it and drafted the manuscript. All authors read and approved the final manuscript.
